# Longitudinal assessment of health-related quality of life and clinical outcomes with at home advanced pneumatic compression treatment of lower extremity lymphedema

**DOI:** 10.1016/j.jvsv.2024.101892

**Published:** 2024-04-16

**Authors:** Frank T. Padberg, Areck Ucuzian, Hasan Dosluoglu, Glenn Jacobowitz, Thomas F. O'Donnell

**Affiliations:** a Department of Surgery, VA New Jersey Healthcare System, East Orange, NJ; b Division of Vascular Surgery, Rutgers New Jersey Medical School, Newark NJ; c Department of Surgery, VA Maryland Healthcare System, Baltimore, MD; d Division of Vascular Surgery, University of Maryland School of Medicine, Baltimore, MD; e Department of Surgery, VA Western NY Healthcare System, Buffalo, NY; f Division of Vascular Surgery, Department of Surgery, State University of New York, Buffalo, NY; g Department of Surgery, VA New York Harbor Healthcare System, New York, NY; h Division of Vascular and Endovascular Surgery, NYU Langone Health, New York, NY; i Division of Vascular Surgery, Department of Surgery, NYU Grossman School of Medicine, New York, NY; j Department of Surgery, Tufts University School of Medicine, Boston, MA

**Keywords:** Lymphatic, Lymphedema, Nonoperative management, Phlebolymphedema, Quality of life, Venous Insufficiency

## Abstract

**Objective:**

This prospective, longitudinal, pragmatic study describes at home treatment with a proprietary advanced pneumatic compression device (APCD) for patients with lower extremity lymphedema (LED).

**Methods:**

Following institutiona review board approval, four participating Veterans Affairs centers enrolled LED patients from 2016 to 2022. The primary outcome measures were health-related quality of life (HR-QoL) questionnaires (lymphedema quality of life-leg and the generic SF-36v2) obtained at baseline and 12, 24, and 52 weeks. The secondary outcome measures were limb circumference, cellulitis events, skin quality, and compliance with APCD and other compression therapies.

**Results:**

Because a portion of the trial was conducted during the coronavirus disease 2019 pandemic, 179 patients had 52 weeks of follow-up, and 143 had complete measurements at all time points. The baseline characteristics were a mean age of 66.9 ± 10.8 years, 91% were men, and the mean body mass index was 33.8 ± 6.9 kg/m^2^. LED was bilateral in 92.2% of the patients. Chronic venous insufficiency or phlebolymphedema was the most common etiology of LED (112 patients; 62.6%), followed by trauma or surgery (20 patients; 11.2%). Cancer treatment as a cause was low (4 patients; 2.3%). Patients were classified as having International Society for Lymphology (ISL) stage I (68.4%), II (27.6%), or III (4.1%). Of the primary outcome measures, significant improvements were observed in all lymphedema quality of life-leg domains of function, appearance, symptoms, and emotion and the overall score after 12 weeks of treatment (*P* < .0001) and through 52 weeks of follow-up. The SF-36v2 demonstrated significant improvement in three domains at 12 weeks and in the six domains of physical function, bodily pain, physical component (*P* < .0001), social functioning (*P* = .0181), role-physical (*P* < .0005), and mental health (*P* < .0334) at 52 weeks. An SF-36v2 score <40 indicates a substantial reduction in HR-QoL in LED patients compared with U.S. norms. Regarding the secondary outcome measures at 52 weeks, compared with baseline, the mean limb girth decreased by 1.4 cm (*P* < .0001). The maximal reduction in mean limb girth was 1.9 cm (6.0%) at 12 weeks in ISL stage II and III limbs. New episodes of cellulitis in patients with previous episodes (21.4% vs 6.1%, *P* = .001) were reduced. The 75% of patients with skin hyperpigmentation at baseline decreased to 40% (*P* < .01) at 52 weeks. At 52 weeks, compliance, defined as use for 5 to 7 days per week, was reported for the APCD by 72% and for elastic stockings by 74%.

**Conclusions:**

This longitudinal study of Veterans Affairs patients with LED demonstrated improved generic and disease-specific HR-QoL through 52 weeks with at home use of an APCD. Limb girth, cellulitis episodes, and skin discoloration were reduced, with excellent compliance.

**Article Highlights**

• **Type of Research:** A multicenter, prospective, single-arm, longitudinal, observational study
• **Key Findings:** Veterans with lower extremity lymphedema were observed for 52 weeks with use of an at home advanced pneumatic compression device. The participants had improved disease-specific and generic quality of life. The outcomes for the secondary end points were decreased limb girth, decreased cellulitis episodes, decreased skin changes, and excellent compliance with compression and device use.
• **Take Home Message:** Improved quality of life and decreased limb girth, cellulitis events, and skin changes accompanied excellent compliance with sustained, at home, adjunctive use of an advanced pneumatic compression device for veterans with lower extremity lymphedema.

Management of lower extremity lymphedema (LED) involves recognition of the pathology, elimination of alternative diagnoses, and the use of compression therapy, comprehensive decompressive therapy (CDT), and appropriate skin care. Compression modalities do not decrease limb swelling but facilitate mobility and prevent limb deterioration during activities of daily living. CDT and manual lymphatic drainage (MLD) are designed to achieve limb reduction and are delivered as an initial therapy with the intent to transition to at home management. Despite its widespread international acceptance, MLD therapy is expensive, subject to insurance constraints, limited in availability, and difficult to sustain; minimal compliance with the at home component limits its effectiveness.^1^^,^^2^ Physical exercise, although providing general health benefits and stimulating lymphatic flow, does not specifically improve lymphatic function or reduce swelling.^1^

A pneumatic compression device (PCD) effectively reduces lower extremity edema and is designed to facilitate therapy at home. A simple PCD provides a predetermined pressure gradient that is not programmable but can have multiple compartments. An advanced pneumatic compression device (APCD) offers a segmental, programmable gradient pressure that can be manually adjusted and tends to have more compartments. The APCD added progressive rhythmic compression waves with improved reduction of lower extremity swelling.^3^, ^4^, ^5^ Previous investigations demonstrated decreased limb girth, decreased limb volume, and decreased frequency of cellulitis events and ulcerations, with symptomatic improvement in mixed lymphedematous extremities.^6^^,^^7^ However, longitudinal health-related quality of life (HR-QoL) evaluation for at home APCD therapy has not been widely studied using current and validated HR-QoL instruments.^1^^,^^2^^,^^8^

Patient-reported outcome measures (PROMs) provide guidance for treatment of chronic, disabling conditions. The primary goal of this investigation is to evaluate the disease-specific and generic HR-QoL for veterans with LED treated with at home APCD therapy. The concomitant secondary goals include measurement of limb edema, cellulitis events, skin response, health care usage (HCU), and compliance (APCD and compression) during a longitudinal 52-week follow-up period.

## Methods

A prospective, multicenter, interventional, postmarket, observational, single-arm, pragmatic study of lower extremity LED was conducted at four Veterans Affairs Health Care Systems to assess the longitudinal benefits of intervention with at home application of an APCD. The first patient was enrolled February 2, 2016, sites were added in 2019, and the study concluded in December 2022. The institutional review board at each Veterans Affairs Health Care System approved the protocol, and each participant provided written informed consent. The study is registered at ClinicalTrials.gov (NCT0266616460; January 22, 2016).

**Fig 1:**
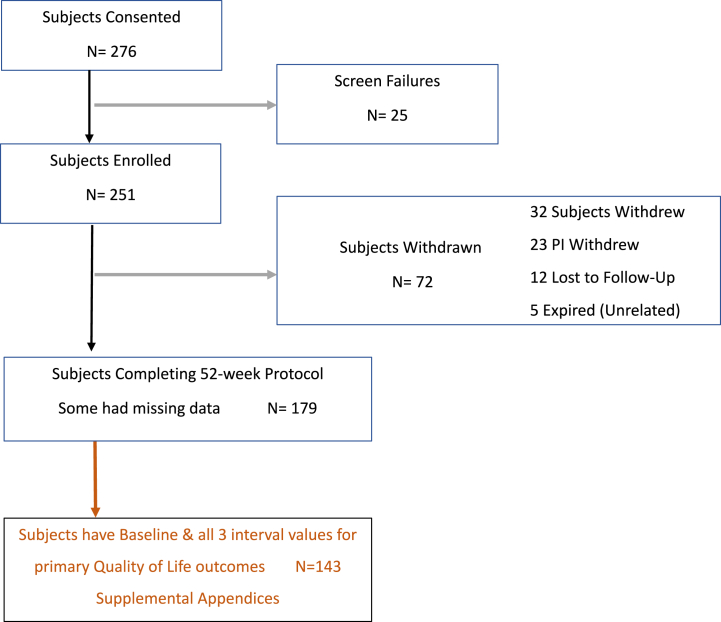
Flow diagram showing inclusion and exclusion of patients. *PI*, Primary investigator.

The 11 study visits were organized during 52 weeks of follow-up. In-person assessments were supplemented by video- and teleconferencing. Remote visits increased in response to the restrictions on in-person clinical encounters imposed by the SARS-CoV-2 pandemic in January 2020, which also affected recruitment. After screening 278 individuals, 251 were enrolled. After 72 were withdrawn, 179 completed the 52-week protocol (Fig 1). An interim report on 74 patients reported improved disease-specific HR-QoL at 52 weeks and in a single SF-36v2 domain at 52 weeks and decreased HCU.^9^

The primary end point included two PROMs measuring HR-QoL: the disease-specific lymphedema quality of life (LYMQOL)-leg questionnaire and the generic short-form 36v2 health survey (SF-36v2; Optum). Both tools were included as online Supplemental Appendexes 2 and 3 in the interim report.^9^ The HR-QoL evaluations were compared at four major visits: at baseline and 12, 24, and 52 weeks.

The LYMQOL-leg was designed and validated for use in patients with lower extremity LED.^10^^,^^11^ It consists of 25 questions with a score scale of 1 to 4 assessing four domains of function, appearance, symptoms, and emotion (mood) and a summary overall score. A decreasing score reflects improvied HR-QoL for the four domains; however, the overall score is scaled as 1 to 10 points, with increasing scores indicating better HR-QoL.

The revised SF-36v2 health survey is validated for evaluating HR-QoL with normative data scoring.^12^ The SF-36v2 consists of 36 questions scaled from 1 to 100 and assessing eight specific domains of physical function, role-physical, bodily pain, general health, vitality, social functioning, role-emotional, and mental health and two summary components (physical and mental). Norm-based scoring algorithms set each scale to the same average of 50 representing a typical healthy U.S. sample and the same standard deviation of 10. Any increase in the SF-36v2score reflects perceived improvement in HR-QoL. Repeat administration of the questionnaire is recommended after >4 weeks for longitudinal outcome studies.

**Supplementary Fig 1:**
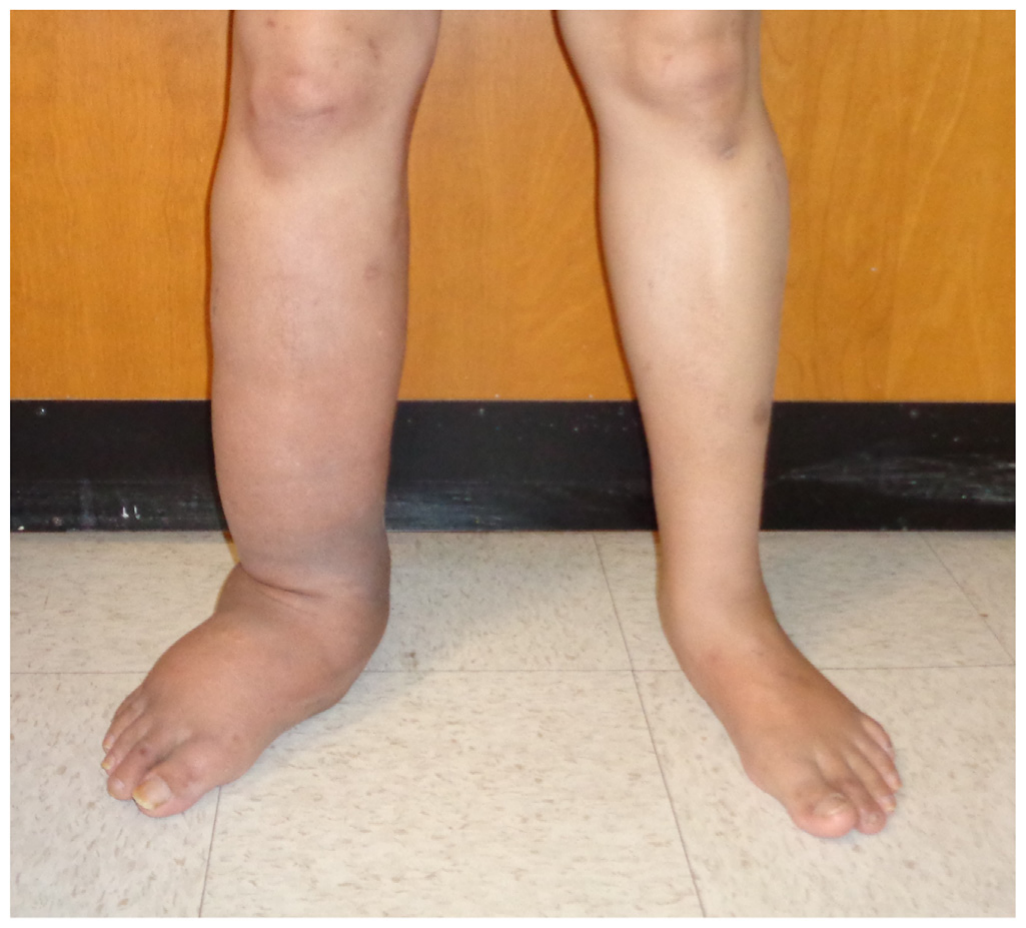
Photograph showing unilateral lymphedematous limb.

The secondary end points of limb circumference and skin changes were also evaluated at baseline and 12, 24, and 52 weeks. Limb photographs were obtained at each of these visits (Supplementary Fig 1). Limb girth measurement of calf circumference was standardized at 18 cm above the floor. The “worst” of the two limbs at baseline was assigned as the study limb. HCU for LED treatment was documented and included self-MLD, compression, skin care, and exercise.

Veterans with a diagnosis of primary or secondary, unilateral or bilateral, LED were eligible to participate if they were aged >18 years, an ability to provide informed consent, and willing to participate in all aspects of the prescribed treatment protocol. The exclusion criteria were as follows: active cancer, active skin or limb infection, recent (prior 6 months) venous thromboembolism, critical limb ischemia, pulmonary edema, heart failure, poorly controlled asthma, previous use of the study APCD, pregnancy, and/or participation in a concomitant drug or device trial.

Patients with a clinical diagnosis of lower extremity LED were screened and enrolled after they completed the informed consent form. LED was classified as primary or secondary. Secondary categories included chronic venous insufficiency, trauma, surgery, cancer, cancer treatment, and/or radiation therapy. Compression garments were provided. The study participants were instructed in routine LED treatment, including the appropriate use of compression garments and proper skin care. The recorded skin changes at each visit included hyperpigmentation, discoloration, hyperkeratosis, dermatitis with eczema, ulceration with blisters, a positive Stemmer sign, squaring of toes, deep creasing at flexion points, papilloma, puffy forefeet, swelling on the dorsum of the foot, increased fat or muscle bulk, and lymphorrhea or weeping edema. Beginning in the latter half of the study, LED was staged from I to III using the International Society for Lymphology staging system. Stage 0 is latent with no swelling. Stage I is soft swelling (pitting) that resolves with elevation. Stage II is spongy swelling (pitting and nonpitting) that does not resolve with elevation; fibrosis might or might not be present. Stage III consists of static elephantiasis where pitting is absent and trophic skin changes develop; extensive fibrotic swelling, blistering, ulceration, lymphorrhea, papilloma, and/or recurrent infections could be present.

### Device specifics

A PCD provides adjunctive therapy for the management of lymphatic dysfunction for either upper or lower extremities. The Flexitouch APCD (Tactile Medical) is clinically proven to stimulate functional improvement of the lymphatic system.^3^^,^^4^ The Flexitouch Plus was introduced late in this trial. It added the ability to treat both legs simultaneously and included enhancements to the garments and controller. Excess fluid is moved from an impaired lymphatic drainage bed to anatomic areas of the body that can absorb and process this fluid. The garment chambers inflate sequentially, with each chamber inflating before the adjacent distal chamber fully deflates. This creates a dynamic wave of therapy that directs fluid into the lymphatic capillaries and maintains distal pressure to prevent backflow.

The patients were provided with a Flexitouch or Flexitouch Plus device and instructed in at home use by trained representatives provided by Tactile Medical. Device-related adverse events were documented. The recommended device engagement of 30 to 60 minutes daily was confirmed during in-person and telephone visits.

### Compliance

Compliance with the APCD and standard compression was monitored at each scheduled visit. Patients kept a diary of APCD use, which was reviewed at each clinic visit, and the level of compliance was entered into the database. Compliance was defined as use of the device or compression for 5 to 7 days per week. Partial compliance was defined as use for only 3 to 4 days per week, and noncompliance as use limited to 0 to 2 days per week. The participants deemed noncompliant were classed as screening failures and withdrawn from the trial at the 4-week visit (Fig 1).

### Study schedule

A telephone visit was conducted at 1 week. In-person visits were scheduled for weeks 4, 8, 12, 24, and 52. Telephone visits were scheduled for weeks 1, 18, 32, and 40. Weight, skin assessment, limb circumference, photographs, and compliance were recorded at each in-person visit. In addition to these, treatment protocols, medications, compliance, adverse events, and device observations were recorded. Both HR-QoL questionnaires were administered at four major visits—baseline (week 0) and weeks 12, 24, and 52. Missed visits are an important consideration in any longitudinal clinical investigation but were substantially exacerbated by the limitations imposed by the SARS-CoV-2 pandemic.

### Statistical analysis

The analysis population included all enrolled participants. Descriptive statistics were calculated for all continuous variables (ie, numbers, mean, standard deviation, median, interquartile range, minimum, and maximum). Frequencies, percentages, and 95% confidence intervals were calculated for categorical data. The data presented are from an analysis of the 179 patients who completed the 52-week protocol, including those with missing interval data. The data for those patients with complete data from all four major visits are also presented but in the Online Supplement..

Data were collected using an electronic data capture system (Clindex) and exported directly into Excel (Microsoft Corp) datasets. The database was built and validated for this study. Study personnel completed the applicable training and were responsible for data entry. The data were exported to an independent statistical consultant who performed the data analysis and prepared the data tables. Tactile Medical clinical research personnel monitored the data acquisition to ensure completeness for missing data. This investigation is reported as recommended in the STROBE (strengthening the reporting of observational studies in epidemiology) guidelines for reporting observational studies.^13^ Analyses were performed using R, version 4.1.0 or higher (R Foundation for Statistical Computing; available at: https://www.R-Project.org).

## Results

Of the 179 patients completing the 52-week follow-up, 163 were men (91%) and 16 were women (8.9%), with a mean age of 66.9 ± 10.8 years. They were moderately obese, with a mean body mass index (BMI) of 33.8 ± 6.9 kg/m^2^. The BMI was <30 kg/m^2^ for 53 patients, ≥30 but <35 kg/m^2^ for 52, and ≥35 kg/m^2^ for 74 patients. Bilateral limb involvement was present in 165 patients (92.2%) (Table I).

**Table I:** Demographic data for 179 patients with a baseline and 52-week visit

Variable	Value
Age, years	
Mean ± SD	66.9 ± 10.8
Median (IQR)	68.4 (61.2-73.4)
BMI, kg/m^2^	
Mean ± SD	33.8 ± 6.9
Median (IQR)	33.1 (29.3-38.4)
Interval from diagnosis to enrollment, years	
Mean ± SD	1.9 ± 4.1
Median (IQR)	0.1 (0.0-1.9)
BMI group, kg/m^2^	
<30	53 (29.6)
≥30 but <35	52 (29.1)
≥35	74 (41.3)
Gender	
Female	16 (8.9)
Male	163 (91.1)
Study leg	
Right	6 (3.4)
Left	8 (4.5)
Bilateral	165 (92.2)
Lymphedema cause	
Primary	17 (9.5)
Secondary	
Cancer	1 (0.6)
Cancer treatment induced	3 (1.7)
Trauma/surgery	20 (11.2)
Other	26 (14.5)
Chronic venous insufficiency or phlebolymphedema	112 (62.6)
ISL lymphedema stage (n = 98)	
I	67 (68.4)
II	27 (27.6)
III	4 (4.1)
Cellulitis, No.	
Patients with previous episodes	21
Patients with episodes during 52-week follow-up	6

*BMI*, Body mass index; *IQR*, interquartile range; *ISL*, International Society for Lymphology; *SD*, standard deviation.

Data presented as number (%), unless noted otherwise.

Primary LED was diagnosed in 17 patients (9.5%) and secondary LED in 162 patients (90.5%). Chronic venous insufficiency or phlebolymphedema was the most common etiology of LED (112 patients; 62.6%). The remainder were attributed to cancer or cancer treatment (4 patients; 2.3%), other (26 patients; 14.5%), and a traumatic or surgical etiology (20 patients; 11.2%).

The ISL stage was available for 98 patients: 67 (68.4%) had stage I, 27 (27.6%) had stage II, and 4 (4.1%) had stage III. Of the 98 patients, 21 (21.4%) experienced one to three episodes of cellulitis during the year preceding enrollment. The diagnosis of LED preceded enrollment by a mean of 1.9 ± 4.1 years. LED and venous HCU events (395) were reported by 104 participants during the year preceding the study, which included clinic, emergency department, and/or walk-in visits (n = 253) hospitalizations (n = 8), and procedures (n = 16).

### Primary outcomes

**Fig 2:**
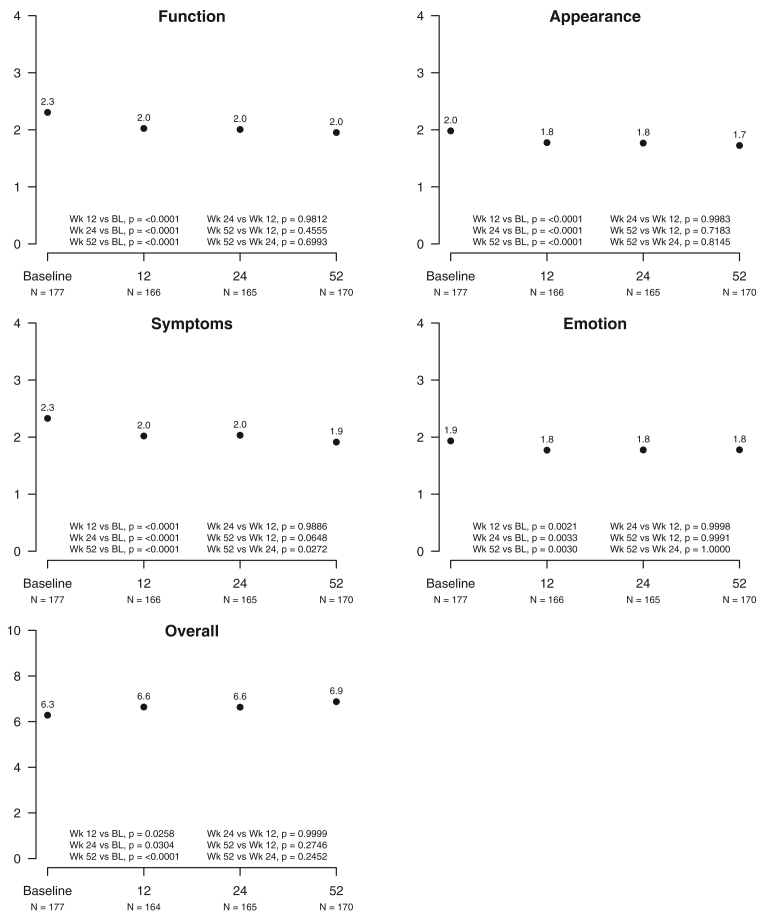
Lymphedema quality of life (*LYMQOL*)-leg scores. Questions are scored from 1 to 4 assessing four domains and a summary overall score. The four domains reflect improved health-related quality of life (HR-QoL) with decreasing scores; however, the overall score is scaled as 1 to 10 points, with increasing scores indicating better HR-QoL. The number of participants reporting was as follows: baseline, n = 177; 12 weeks, n = 166; 24 weeks, n = 165; and 52 weeks, n = 170.

**Supplementary Fig 2:**
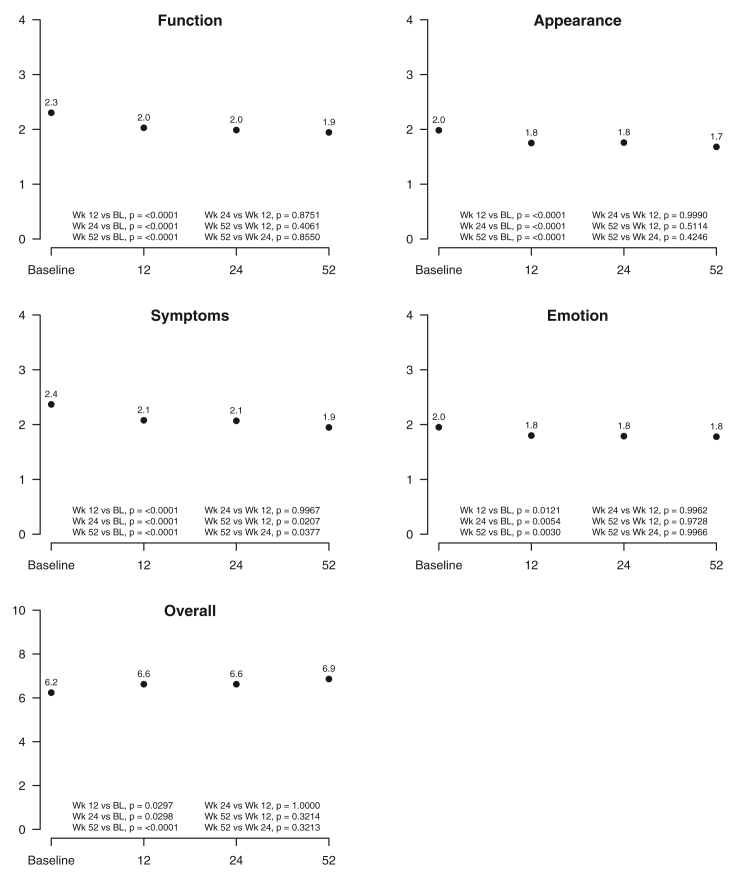
Lymphedema quality of life (LYMQOL)-leg. Patients with data from all four major visits recorded (n = 143).

After 12 weeks, the participants reported improvement in all four LYMQOL-leg domains of function, appearance, symptoms, and emotion and the overall summary score, with the improvement sustained through 52 weeks (*P* < .0001). The findings did not differ between those with missing data (Fig 2) and those with data from all four major visits (Supplementary Fig 2). There were no differences in the 52-week scores in any domain between the BMI categories, limb girth, ISL stage 0 to I vs II to III, or a history of cellulitis. Although validated for use for lower limb LED, normative comparisons are not currently available for LYMQOL-leg.^10^^,^^11^

**Fig 3:**
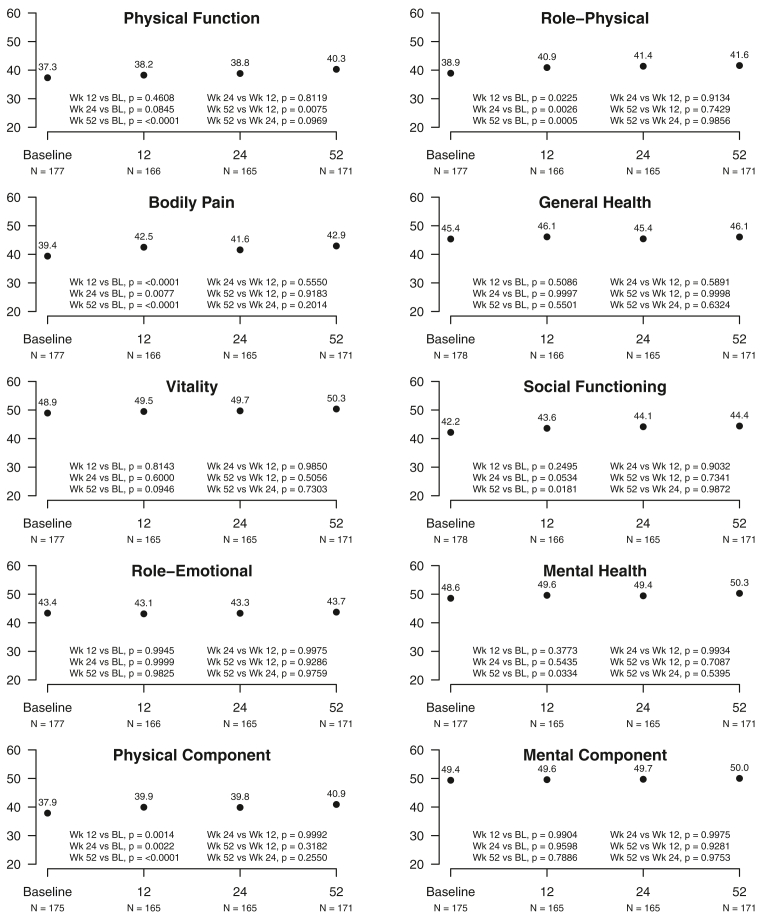
SF-36v2 (Optum). The SF-36v2 consists of 36 questions scored from 1 to 100 for eight specific domains and two summary components (physical and mental). The SF-36v2 scores increase with perceived improvement in health-related quality of life (*HR-QoL*) The number of participants reporting was as follows: baseline, n = 178; 12 weeks, n = 166; 24 weeks, n = 165; and 52 weeks, n = 171.

**Supplementary Fig 3:**
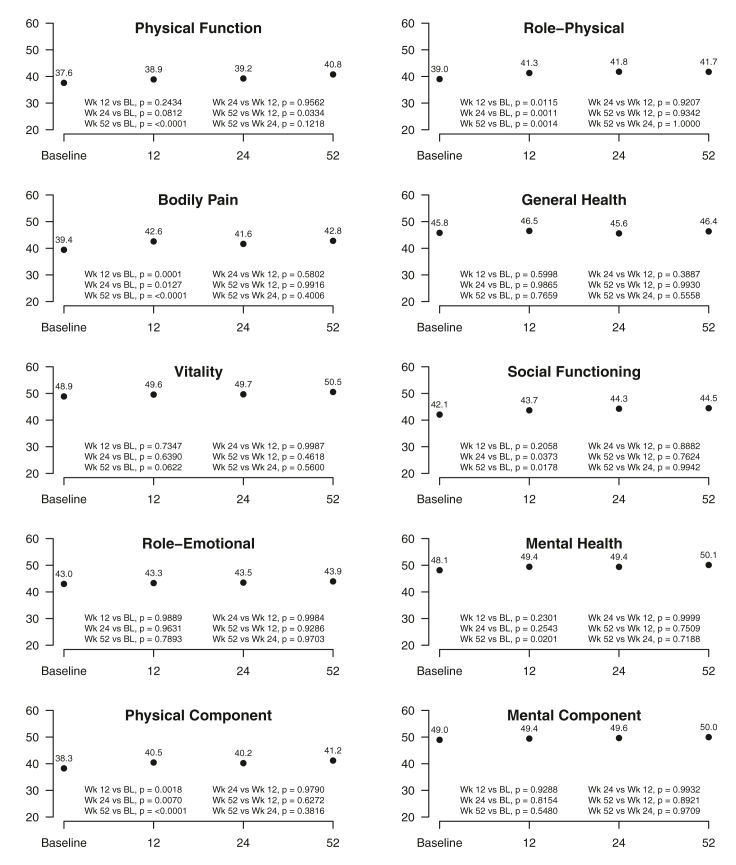
SF-36v2 (Optum). Patients with data from all four major visits recorded (n = 143).

SF-36v2 scores of <40 for physical domains indicate substantial reductions in HR-QoL compared with the typical U.S. population. Significant improvement compared with baseline was observed in six domains. Three SF-36v2 domains were improved at 12, 24, and 52 weeks: role-physical (*P* < .02-.0006), bodily pain (*P* < .0077-.0001), and physical component (*P* < .0014-.0001). At 52 weeks, three additional domains of physical function (*P* < .0001), social functioning (*P* < .0181), and mental health (*P* < .0333) had improved HR-QoL. No differences were identified in the four domains of general health, vitality, role-emotional, or mental component (Fig 3). The findings did not differ between those with missing data (Fig 3) and those with complete data (all four visits), except for social functioning in the latter group at 24 weeks (*P* < .0373; Supplementary Fig 3).

### Secondary outcomes

#### Reduction in limb girth

**Fig 4:**
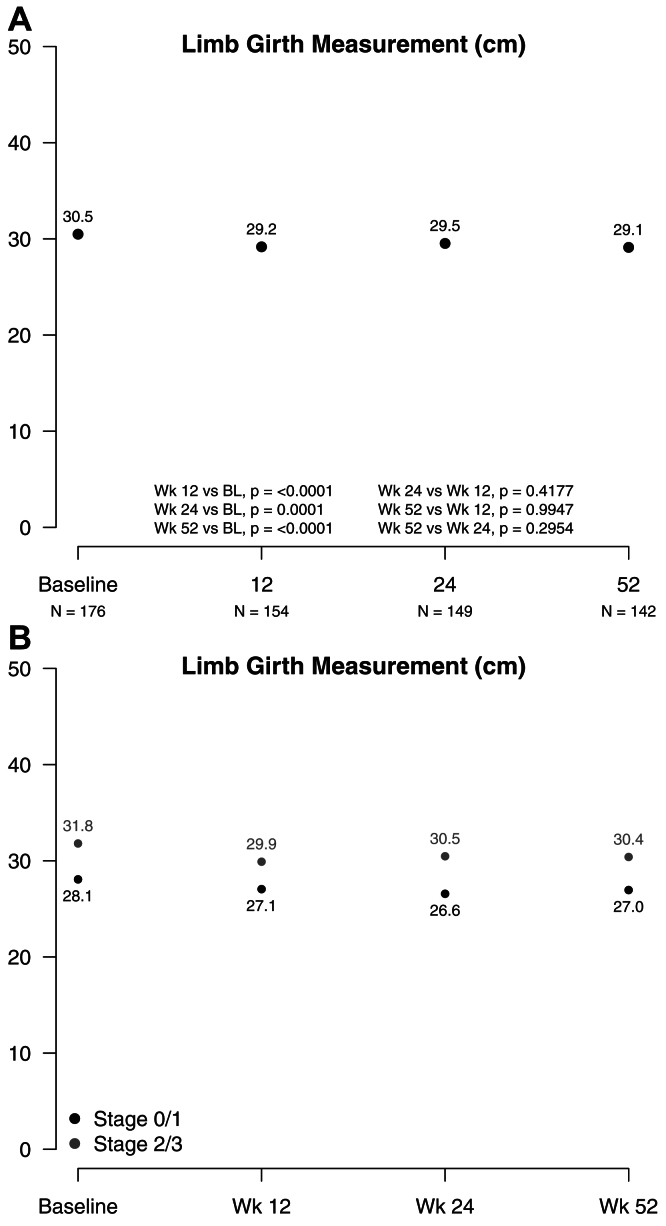
Limb girth measurements. Calf circumference was measured at 18 cm above the floor. The “worst” of the two limbs at baseline was assigned as the study limb after completion of the clinical data form. The number of patients reporting was as follows: baseline, n = 176; 12 weeks, 154; 24 weeks, n = 149; and 52 weeks, n = 142.

**Supplementary Fig 4:**
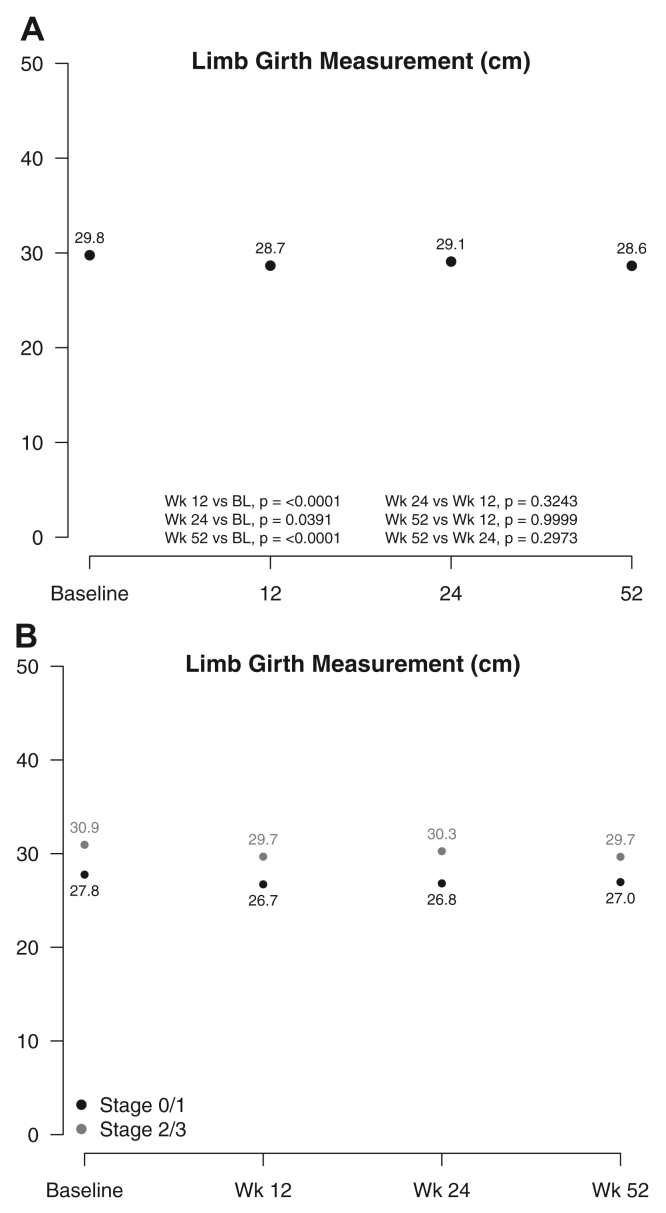
Limb girth (secondary end point). Patients with data from all four major visits recorded (n = 121).

Compared with that at baseline, the mean limb girth measurements in the 179 patients with missing postbaseline data points decreased 1.4 cm (*P* < .0001) at 52 weeks. Girth was significantly different compared with baseline at the 12-, 24-, and 52-week study visits. The mean limb girth at 52 weeks decreased by 1.2 cm (*P* < .0001) in the 121 patients with data from all four major visits. None of the interval mean girth measurements were different from their immediately preceding measurement in either group. The statistical comparisons were similar between those with missing data (Fig 4) and those with data from all four major visits (Supplementary Fig 4).

The mean limb girths for those with ISL stages II and III were greater than those with stage 0 and I at baseline (*P* < .0001) and at 52 weeks (*P* < .0051). The mean stage 0 and I limb girth decreased by 1.1 cm from baseline at 52 weeks. The mean stage II and III limb girth decreased by 1.4 cm from baseline. For the 121 patients with all four measurements, the decrease was 0.8 cm and 1.2 cm for the stage 0 and I and stage II and III limbs, respectively. The greatest decrease in limb girth was 1.9 cm at 12 weeks (6%) in the ISL stage II and III group with missing data (Fig 4).

#### Reduction in cellulitis events

In the year preceding enrollment, 21 participants had a history of medical encounters for cellulitis. Seven events in six patients were recorded during the 52-week trial. This represented a reduction from 21.4% to 6.1% in cellulitis events (*P* < .0011).

#### Reduction in skin abnormalities

Although skin abnormalities in the lymphedematous limb will often be the focus for an individual patient, the number of patients with recorded skin assessments decreased substantially over time, resulting in wide 95% confidence intervals, and was not amenable to accurate statistical evaluation. Interesting observations are listed with the baseline and week 52 percentages. Hyperpigmentation/discoloration decreased from 75.4% to 40%. Ulceration/blisters decreased from 20.3% to 14.1%. Papilloma decreased from 5.8% to 2.4%. Finally, lymphorrhea decreased from 8% to 1.2%.

#### Outstanding compliance with APCD and compression therapy

Compliance was recorded for all 179 participants and defined as use of the device for ≥5 to 7 days per week for 1 hour (Table II). One week after instruction on use of the APCD, 83.3% of the participants were compliant. The best APCD compliance was 92% at the 8-week visit. At the 40-week telephone visit, 67.2% were fully compliant. Partial compliance (3-5 days per week) in week 1 was 10.3% and had increased to 21.3% in week 40. Those who became noncompliant (0-2 days per week) during the trial comprised the remaining 11.5% at week 40. At week 52, 71.9% remained fully compliant with the APCD.

**Table II:** Compliance with compression and advanced pneumatic compression device (*APCD*)

Variable	Baseline	Week 4	Week 12	Week 24	Week 52
Compression therapy, No.	179	170	166	166	179
Self-MLD	9 (5; 2.7-9.3)	10 (5.9; 3.2-10.5)	10 (6.0; 3.3-10.7)	12 (7.2; 4.2-12.2)	15 (8.4; 5.1-13.4)
Compression garments	115 (64.2; 57.0-70.9)	117 (68.8; 61.5-75.3)	119 (71.7; 64.4-78.0)	121 (72.9; 65.7-79.1)	133 (74.3; 67.4-80.1)
Compression bandages	15 (8.4; 5.1-13.4)	13 (7.6; 4.5-12.6)	7 (4.2; 2.1-8.4)	6 (3.6; 1.7-7.7)	11 (6.1; 3.5-10.7)
ACTitouch	6 (3.4; 1.5-7.1)	14 (8.2; 5.0-13.3)	14 (8.4; 5.1-13.7)	13 (7.8; 4.6-12.9)	11 (6.1; 3.5-10.7)
Any^a^	123 (68.7; 61.6-75.1)	128 (75.3; 68.3-81.2)	123 (74.1; 66.9-80.2)	126 (75.9; 68.9-81.8)	140 (78.2; 71.6-83.6)
APCD therapy, No.	179	108	111	114	139
Compliant 5-7 d/wk	NA	99 (91.7; 84.9-95.6)	95 (85.6; 77.9-90.9)	97 (85.1; 77.4-90.5)	100 (71.9; 64.0-78.7)

*MLD*, Manual lymphatic drainage; *NA*, not applicable.

Data presented as number (%; 95% confidence interval), unless noted otherwise.

a Including all garments, bandages, and ACTitouch (ACTitouch is an ambulatory pneumatic compression system classified as a simple pneumatic compression device, which was used on a limited basis as an integral component of lymphedema care using compression much the same as compression hosiery and MLD).

Compliance with any compression, including garments, bandages, ACTitouch, and/or self-administration of MLD was 78.2% at 52 weeks; a significant increase from baseline of 68.7% (*P* < .04). There was an increase in use for each modality except for compression bandages, which decreased from 8.4% to 6.1%. Between baseline and 52 weeks, self-MLD use increased from 5% to 8.4%, compression garment use increased from 64.2% to 74.3%, and ACTitouch use increased from 3.4% to 6.1%.

Two minor, nonserious, device-related events were reported (ie, self-limited “ankle pain”). There were no device- or procedure-related deaths in this study population.

### Health care usage

Although HCU for LED and venous-related events during the study (n = 279) were less than the 395 experienced during the year prior, the difference was not statistically significant. These included clinic visits (n = 236), procedures (n = 16), emergency department, walk-in, urgent care visits (n = 11), telehealth visits (n = 8), and hospitalizations (n = 8).

Complications from chronic LED frequently precipitate episodes of HCU. Although reasonable to expect that HCU would be decreased with compliant LED management, this was not observed in the current report for this 52-week supervised protocol.

## Discussion

Adjunctive use of an APCD for patients with lower extremity LED was accompanied by improved HR-QoL as determined by both disease-specific and generic measures. The limb girth decreased within months of enrollment and was sustained throughout the study. Patient-reported cellulitis events during the year preceding enrollment were decreased during the year of the study. Compliance with both compression therapies was outstanding, with both compression garments and the APCD. A predominance of phlebolymphedema with mixed etiologies is consistent with other work noting that malignant disease is not the most common etiology of lower extremity LED.^2^^,^^6^^,^^14^

PROMs measuring HR-QoL during LED therapy have not been widely studied, and disease-specific instruments have only recently been developed.^8^^,^^15^ Several investigations deployed venous instruments to assess HR-QoL. The systematic review by Müller et al^16^ identified two randomized controlled trials that found no difference in QoL between MLD and controls for patients with mixed lower extremity LED. Muluk et al^17^ used a nonvalidated, five-question “self-reporting” form after initiating APCD therapy. Blumberg et al^6^ used a venous QoL tool (CIVIQ-2) to report symptom improvement in patients with lower extremity LED. In contrast, a disease-specific HR-QoL instrument was used for our investigation.

The LYMQOL questionnaire, introduced in 2010, is validated for use in both upper and lower extremity LED.^10^ Improved disease-specific HR-QoL was demonstrated by 12 weeks in all five elements of the LYMQOL-leg instrument. The narrow spread of a 4-point scale produces less dispersion in raw numerical values; however, statistical significance can be observed in studies with large populations such as ours. Lim et al^8^ noted a second upper extremity HR-QoL tool but no others for the lower extremity. Although upper extremity LED is more commonly a consequence of breast cancer therapy, many of the theoretical constructs (ie, MLD, compression, pneumatic compression) associated with management are also applicable to the lower extremity.

As a generic instrument, the SF-36v2 might not be responsive to disease-specific conditions. However, the SF-36v2 has the advantage of normative data scoring.^12^ LED is a chronic debilitating condition and produced several baseline scores <40—a standard deviation less than the normative value of 50 for typical healthy U.S. group samples. The results illustrate the adverse effects of LED on the physical components of health. The interim report from this study reported improvement in a single domain (physical component) of the SF-36v2 at 52 weeks compared to baseline.^9^ In contrast, in the present report, we demonstrate improvement in 6 of the 10 domains. Improved HR-QoL was observed at 12, 24, and 52 weeks for the domains of role-physical, bodily pain, and physical component. The domains of physical function, social functioning, and mental health were improved at 52 weeks. Instruments measuring longitudinal changes in HR-QoL provide support for LED treatment.

Decreased limb girths were sustained throughout the 52 weeks and correlated with improved HR-QoL scores. Previous work with this APCD also demonstrated a decrease in the calculated limb volume.^17^

The cutaneous physical manifestations associated with increasing ISL stage are a major component of the impact of LED on an individual's HR-QoL. Decreased swelling and a reduced frequency of cellulitis episodes are clinical manifestations of a successful comprehensive treatment approach.

The interim report from this trial reported “…an overall reduction in lymphedema-related clinic visits, urgent care use, and hospitalization.”^9^ However, the optimistic conclusion that HCU was decreased at 2 months was not sustained.^9^ Although other investigations have demonstrated decreased LED-related health costs with use of an APCD,^17^^,^^18^ this was not observed in this VA clinical protocol.

During this 52-week observation, remarkable compliance with both mechanical and standard compression therapy was recorded. Although the positive effects of frequent reminders imposed by the 11-visit protocol cannot be separated from the effects of the APCD, the clinical responses to comprehensive LED therapy are encouraging. A recent longitudinal study from a comprehensive lymphedema treatment center suggested compliance with lymphedema therapy was improved with the addition of a PCD. At a median 18-month follow-up, compliance with the PCD was 84% compared with only 53% for those without the PCD.^19^

The current report of this VA trial offers more robust HR-QoL data from all major visits, with more than double the number of study participants. Valuable details on the secondary outcomes include reduced girth, decreased cellulitis, and outstanding compliance.

Chronic edema is “synonymous with the presence of lymphedema, inasmuch as all edema represents a relative lymphatic drainage failure.”^20^ LED is a clinical diagnosis. Cure is not anticipated or expected. Limb elevation, in conjunction with the mechanical adjunct of an APCD, work in combination to effect a reduction in limb edema. Debate regarding the differences between various APCDs and PCDs is beyond the scope of this report; however, the effectiveness of compression devices has been demonstrated in numerous studies.^1^^,^^2^^,^^6^^,^^7^^,^^9^^,^^17^, ^18^, ^19^^,^^21^ Direct evidence of improved lymphatic function has been demonstrated with the specific APCD used in this protocol.^3^^,^^4^ A high use of compression garments is important for maintaining a decreased limb volume.

An increasing BMI is associated with decreased lymphatic function in an obese population such as this.^14^^,^^19^^,^^22^, ^23^, ^24^ The most debilitating symptoms associated with chronic venous insufficiency and phlebolymphedema have responded to weight loss.^25^ Concomitant venous obstruction or reflux can contribute to these symptoms; however, in patients with a high BMI, venous interventions might have less impact on the outcomes.^26^ Millen et al^27^ recently reported primary popliteal venous valvular dysfunction with increased obesity. Thus, obesity itself appears to make a significant contribution to venous–lymphatic insufficiency. Obesity should be considered an etiology and is unique in offering increasing potential for reversibility.

Consensus recommendations recommend adjunctive use of pneumatic compression for patients with lower extremity LED.^1^^,^^2^^,^^21^ Standard practice includes elevation of the limb when feasible, external compression garments, early treatment of cellulitis, and appropriate skin care. Maximizing function and mobility become critical determinants of patient satisfaction. CDT and MLD are normally initiated as supervised therapy with the intent of transitioning to self-management within several weeks. However, self-MLD is difficult to accomplish (especially in the lower extremity), time-consuming, expensive, and subject to insurance constraints. Although CDT has been widely advocated, there is a paucity of objective data supporting its use.^8^^,^^16^^,^^28^ The experience in this trial is consistent with this clinical reality, because only 5% to 8% of our participants used self-MLD. The APCD is designed to accomplish a reduction in edema and offers ease of use for daily application in the home.

### Study limitations

The pandemic resulted in premature cessation of enrollment and an inability to conduct in-person visits for measurements and supervision of questionnaire completion. Thus, the study coordinators were not able to obtain all interval study visit data for the 179 participants who completed the 52-week observation. Data analysis for those with random missing data and from missed interval visits were analyzed separately from those with data for all four major visits. There was no difference in the statistical evaluation of the findings between the analyses. It is difficult to separate the effects of frequent monitoring on the responses and compliance. The male predominance of the VA population could reduce generalizability.

## Conclusions

In this longitudinal 52-week study, patients experienced significant improvement in their disease-specific and generic HR-QoL with the use of an at home APCD as early as 12 weeks. The decreased limb girth within 3 months was sustained through the 52-week observation period. Cellulitis events decreased, although overall HCU did not. Compliance of up to 91% with the prescribed therapy was excellent. These findings support the adjunctive use of the APCD for patients with lower extremity LED.

We thank the Tactile Medical clinical support team and Jake DuFresne and Marc Schwartz of MS Biostatistics, LLC, for statistical analysis.

## Author Contributions

Conception and design: FP, GJ, TO

Analysis and interpretation: FP, AU, HD, GJ, TO

Data collection: FP, AU, HD, GJ, TO

Writing the article: FP, AU, HD, GJ, TO

Critical revision of the article: FP, AU, HD, GJ, TO

Final approval of the article: FP, AU, HD, GJ, TO

Statistical analysis: Not applicable

Obtained funding: TO

Overall responsibility: FP

## Funding

The authors received funding from Tactile Medical to conduct this study, and T.F.O. was a consultant whose engagement ended on May 31, 2023.

## Disclosures

None.
